# A qualitative evaluation of access to essential laboratory services for communicable diseases at the primary health care level in the Western Pacific Region

**DOI:** 10.1186/s41182-025-00797-3

**Published:** 2025-11-07

**Authors:** Innocent Mupunga, Wayne Dimech, Kiyohiko Izumi, Kalpeshsinh Rahevar, Kazim Sanikullah, James F. Kelley, Fukushi Morishita, Huong Tran, Rajendra P. Yadav

**Affiliations:** 1 National Serology Reference Laboratory, Fitzroy, VIC 3065 Australia; 2 World Health Organization Regional Office for the Western Pacific, Manila, Philippines

**Keywords:** Primary healthcare, Laboratory, Communicable diseases, Diagnostics, Quality assurance, Testing

## Abstract

**Background:**

Availability and access to quality laboratory diagnostics at the primary healthcare (PHC) level are critical to achieving universal health coverage. However, significant access disparities still exist. This evaluation aimed to understand the current laboratory capacity and infrastructure for communicable diseases testing at the PHC level and identify systemic challenges affecting access.

**Methods:**

This evaluation was conducted in eight low-middle-income countries (Cambodia, China, Lao PDR, Malaysia, Mongolia, PNG, Philippines, and Solomon Islands) in the WHO Western Pacific Region. Data were collected by reviewing existing WHO and country-level policies, guidelines, and reports on laboratory services for communicable diseases at the PHC level, as well as virtual interviews with participants at various levels of healthcare.

**Results:**

Most countries are progressing well towards improving laboratory access at all levels. Activities contributing to improved access include point-of-care testing, integrated sample transport systems to facilitate referral of samples, community engagement, and efforts towards combating stigma and discrimination. Vertical disease programs supported by development partners bridge the funding and capacity gaps for several high-priority public health problems, but these support streams are dynamic and often diminishing. The systemic challenges identified were categorized into three thematic areas: (1) weaknesses in primary healthcare systems; (2) limited community and individual engagement; and (3) persistent socio-economic barriers. Potential solutions and recommendations should include a stepwise approach customized for each country’s context in collaboration with all stakeholders.

**Conclusion:**

Despite the progress already achieved, most countries in the region still face significant challenges in improving access to essential laboratory services for communicable diseases at the PHC level.

**Supplementary Information:**

The online version contains supplementary material available at 10.1186/s41182-025-00797-3.

## Introduction

Strong primary healthcare (PHC) systems are vital for achieving universal health coverage (UHC), the ambitious goal of reaching the full range of quality health coverage without financial hardship [1]. Quality PHC systems are the most inclusive, equitable, cost-effective, and efficient approach for addressing the public health challenges posed by communicable diseases, noncommunicable diseases (NCDs), aging populations, and reaching marginalized populations [1]. Availability and access to affordable healthcare that includes quality laboratory diagnostics, health education and promotion, and disease prevention activities at PHC is a critical component of UHC programs and empowers communities and individuals [2]. Quality diagnostic systems contribute towards effective disease detection, surveillance, prevention and treatment [3]. During potential pandemics, accurate and timely diagnosis facilitates contact tracing, early detection of cases, the implementation of a cascade of care, and the easing of any pandemic-induced restrictions.

Significant disparities in access to essential laboratory services exist in the Western Pacific Region between and within countries [4–6]. Availability of testing depends on factors such as economic development, infrastructure, and public health priorities [7]. High-income countries have well-established networks of public and private laboratory services with advanced technologies, skilled personnel, and comprehensive testing capabilities [5, 8, 9]. In contrast, low- and middle-income countries (LMIC) face resource and infrastructure constraints, have limited laboratory capacity and inadequate staff training [5, 10]. The situation is even more challenging in small Pacific Island countries [5, 10–13].

These challenges are compounded at the PHC level, especially in rural and remote areas where the marginalized, socially disadvantaged, and hard-to-reach populations live [14]. PHC facilities usually lack basic laboratory infrastructure, equipment, trained personnel, and essential supplies needed to conduct tests effectively [1]. As a result, patients may travel long distances or wait extended periods to access laboratory services, delaying diagnosis and treatment.

PHC facilities support public health programs like disease screening, diagnosis, and surveillance. These programs are supported by international development organizations and bilateral partners, providing technical assistance, capacity-building support, and guidelines to support training, improving laboratory infrastructure, supply chain management, and implementation of quality assurance (QA) systems [7, 15]. Each public health program often assesses laboratory services to identify program-specific challenges, focusing on higher-tier laboratories. While higher-tier laboratories may receive attention, the intricate dynamics and constraints of PHC-level laboratories remain largely unexplored, impeding the development of comprehensive and system-wide solutions to enhance the accessibility and effectiveness of diagnostic services in underserved areas.

This evaluation aimed to identify access status, understand current laboratory capacity and infrastructure for communicable disease testing and identify systemic challenges affecting access to these laboratory services at the PHC level.

## Methodology

The evaluation was conducted from May–September 2023. Ten countries were initially selected by WHO, and these were approached through their respective WHO Country offices (WHOCO). Of these, eight countries confirmed their availability and willingness to participate in this study. Subject matter experts were drawn from the following participating countries: Cambodia, China, Lao People’s Democratic Republic (Lao PDR), Malaysia, Mongolia, Papua New Guinea (PNG), Philippines and the Solomon Islands.

### Selection of participants

Participants included technical representatives from WHO Country Offices (WHOCO) and Western Pacific Regional Office; national and provincial health officers; frontline workers; community/patient representatives; and representatives of community-based organizations (CBO), non-governmental organizations (NGO) and faith-based organizations (FBO). They were selected by the Ministry of Health (MOH) focal points in consultation with the WHOCO. The number of study participants from each country or organization is shown in Table 1.

**Table 1:** Study participants from each country or organization

Participants	Country or organization								
	WHO WPR	Cambodia	China	Lao PDR	Malaysia	Mongolia	PNG	Philippines	Solomon Islands
WHO RO	7								
WHO CO		2	2	4	2	2	2	2	2
National		4	1	1	4	2	2	2	
Provincial		2	5	2	2				
Frontline		4		1	2				
Community or NGO		4				3		2	

*WHO WPR* WHO Western Pacific Regional Office, *WHO RO* WHO Regional Office experts, *WHO CO* WHO Country Office experts, *National* national level health officers, *Provincial* provincial-level health officers, *Frontline* frontline health officers, *Community or NGO* community leaders, patients or NGO staff

### Data collection and analysis

Data collection was through a literature review and virtual in-depth interviews with participants at various levels of healthcare.

#### Literature review

A literature review was conducted to examine existing WHO and country-level policies, guidelines, and reports on laboratory services for communicable diseases such as human immunodeficiency virus (HIV), hepatitis B and C, tuberculosis (TB), malaria, and syphilis at the primary health care (PHC) level. The review focused on key aspects including the structure and accessibility of laboratory services, quality assurance mechanisms, and disease surveillance systems. WHO policies and guidelines were sourced from the official WHO website and through consultations with WHO technical experts across relevant infectious disease areas. Country-specific reports, policies, and guidelines were obtained via WHOCO and Ministry of Health (MOH) focal persons.

#### Virtual interviews

An initial virtual meeting was held with the WHOCO technical representatives to explain the project’s purpose and the country office’s role and to interview them. The WHOCO then coordinated with the MOH focal persons to select participants at each of the healthcare levels. The interviews were conducted in English, and language interpretation services were provided by WHOCO or hired professional translators, where required. MOH focal persons conducted interviews for community/patient representatives in the local language.

Interviews were structured around four semi-structured interview guides (see Supplementary material) that were specifically designed for each stakeholder group in collaboration with the experts from the WHO Western Pacific Regional Office. The interview guides focused on the status and challenges affecting availability, accessibility, affordability, acceptability, adequacy, and quality of the laboratory system. The interview guides used open-ended questions to encourage participants to share their thoughts in depth and also allowed flexibility to ask probing and follow-up questions as themes emerged during the interviews. The responses were evaluated at the country level and not at the individual participant level.

The information collected from literature reviews and interviews was analysed and findings were categorized by themes.

### Ethical considerations

Each of the subject matter experts was selected and authorized by the relevant MOH to participate in the evaluation. All participants provided verbal informed consent; they were free to withdraw from the evaluation at any time, and confidentiality was ensured throughout the evaluation.

## Results

**Fig. 1:**
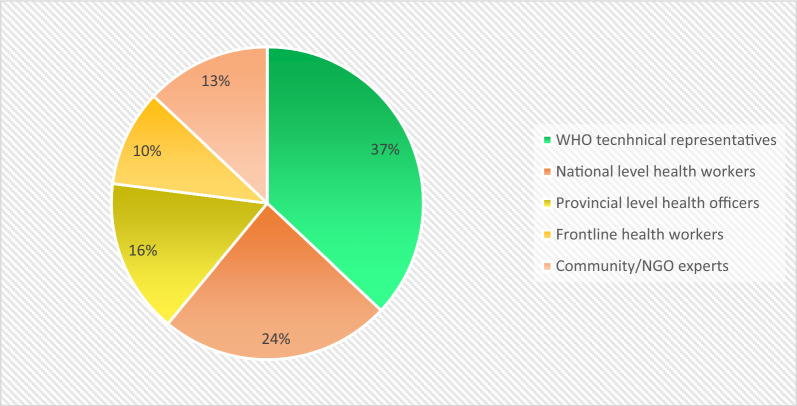
Distribution of participants

A total of 68 experts from the eight countries participated in this evaluation. The distribution of participants is shown in Fig. 1.

**Fig. 2:**
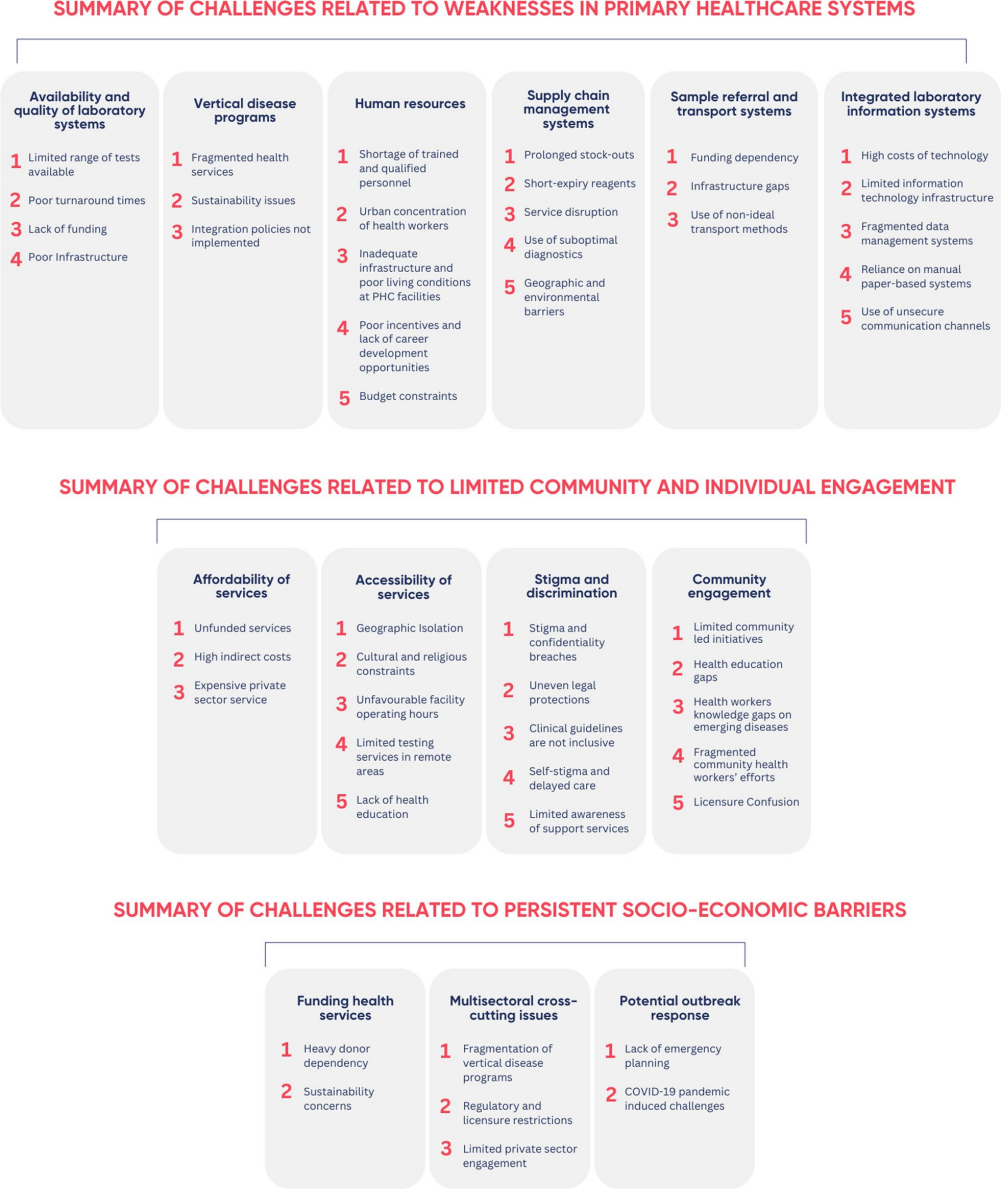
Summary of the challenges identified under each thematic area

The findings of this evaluation were classified into three thematic areas: (1) weaknesses in PHC systems; (2) limited community and individual engagement; and (3) persistent socio-economic barriers. A summary of the challenges identified under each thematic area is shown in Fig. 2.

### Weaknesses in PHC systems

1. Availability and quality of laboratory systems

All participating countries were working towards expanding the availability of testing services, especially for communicable diseases. For example:

- China: screening tests for most communicable and NCDs are available at 95% of the lower tier levels with an effective sample referral system for confirmatory testing at the county or city level. Results for the referred tests are available electronically within 12–48 h.
- Malaysia: approximately 70% of the PHC clinics in the country have laboratory services.
- PNG: testing capacity at the PHC level was improved using point-of-care (POC) tests, equipping more test sites with new testing technologies (e.g., GeneXpert), and training more microscopists.
- Mongolia, Lao PDR, Cambodia, and Solomon Islands: WHO-prequalified POC and rapid tests have expanded PHC services, with reactive tests referred for confirmatory testing and good turnaround times. One frontline health worker reported that “*…confirmatory results for clients are available within 1–2 days.*”

Some countries reported that testing service quality is monitored through internal and external QA activities, for example:

- PNG: QA for infectious diseases is managed by the Central Public Health Laboratory.
- Philippines: QA performance is tied to licensure; satisfactory performance is a prerequisite. Non-complying laboratories lose their licenses, and licensure is only re-instated after corrective action.
- Malaysia: QA activities including review of malaria and TB slides and QA programs for microbiology, clinical biochemistry, and haematology are overseen at the national level. To support the quality of services for PHC clinic laboratories, a national standard based on ISO 15189 was developed.

Despite the positive efforts to expand the availability and quality of laboratory systems, some challenges were reported:

- Limited range of tests available: seven countries reported that PHC facilities mostly offer basic POC and rapid diagnostic tests, and vertical disease program-related testing. Some tests are often restricted to pregnant women or symptomatic patients (e.g., hepatitis testing).
- Poor turnaround times: four countries noted delays in test results for samples referred to district or provincial hospitals. One provincial-level expert commented, *“The sub-optimal testing coverage leads to the massive practice of diagnosis without laboratory testing.”*
- Lack of funding: five countries noted funding constraints for QA, and supervision and monitoring of PHC facilities by higher-tier laboratories. Resources are often prioritized for “lifesaving” products, leaving gaps in support services. One national-level expert noted that “*the limited funding is sometimes directed to ‘lifesaving’ activities/products and few resources left for other important behind the scenes activities. For example, the bulk of the Global Fund budget prioritized the ‘lifesaving’ products such as drugs and test kits, leaving out other critical activities such as internal and external QA, staff training and supervision.*”
- Poor infrastructure: four countries reported that many lab facilities are outdated, unsafe, and lack adequate space to accommodate essential equipment or workflows. Intermittent supplies of water and electricity are common, especially in rural areas, severely hampering availability and quality of testing. In one country, approximately 10% of health centres closed during the last 5 years due to the poor condition of facilities, and/or lack of staff housing. A national level health expert reported that “*For example, in 2021, 22% of health facilities were without water and 49% were without functioning toilets.*”

2. Strengthening integration of vertical disease programs

Some countries reported that vertical disease programs have efficient systems and processes, and better funding for staff training, QA processes, supervisory visits, and reagents procurement. Ongoing efforts to integrate vertical health programs into existing health systems to offer holistic patient service were also noted:

- Solomon Islands: GeneXpert machines procured for COVID-19 testing are now being repurposed for TB testing.
- Philippines: efforts are ongoing to integrate TB programs with other healthcare programs. These programs often have efficient sample transportation systems and backup mechanisms for equipment failures, which can also support and enhance broader PHC service delivery. It was reported that “*…if one GeneXpert machine in one municipality breaks down, they have an existing arrangement to refer specimens to the nearest municipality with a GeneXpert that is running*.”
- PNG: a diagnostic network optimization exercise was recently conducted to visualize and improve diagnostic capacity and access across programs through an improved diagnostic network set-up.

Five countries reported challenges with the vertical disease programs.

- Fragmented health services: most programs are funded and managed in silos parallel to the general health services worsening the shortage of human resources for laboratory work in limited resource countries. These programs usually implement robust disease-specific activities that are not cross-cutting and leading to fragmented health services. For example, monitoring HIV treatment might require testing that is not funded as part of the disease-specific programs (e.g., kidney or liver function tests). This leaves patients at risk of paying high out-of-pocket costs for additional testing.
- Sustainability issues: it was also reported these disease-specific programs lack sustainability as established activities are not continued without program funding. Many effective systems (e.g., sample referral for TB/HIV) are funded by external donors like the Global Fund. Without government takeover and funding, these systems risk discontinuation after donor support ends.
- Integration policies not implemented: some countries face implementation challenges for health programs’ integration policies because most disease programs do not fund additional services required for integration, and most governments lack the budget to fund the services. For example, during the COVID-19 pandemic, one country received PCR machines for their provinces from development partners without equipment maintenance support resulting in extended downtimes when equipment failed.

3. Human resources

All the countries assessed reported a need for more trained and qualified laboratory, nursing, and other support personnel at all levels of health services, particularly at the lower tiers of the laboratory systems. The following issues were commonly reported:

- Shortage of trained and qualified personnel, including laboratory professionals, nurses, and other support staff, especially in rural and remote areas.
- Urban concentration of health workers: one country reported that over 50% of health workers were based in the capital, even though 81% of the population lived outside the capital.
- Inadequate infrastructure and poor living conditions at PHC facilities deter qualified staff from accepting PHC postings and contribute to the urban concentration of health workers.
- Poor incentives and lack of career development opportunities contribute to staff preference for urban or higher-level facilities.
- Budget constraints: some countries noted that insufficient human resources funding prevented the hiring of new personnel, leading to reliance on unpaid volunteers waiting for job openings, whose availability and reliability were inconsistent.

4. Supply chain management

Some countries have adopted centralized procurement models to streamline supply replenishment and improve efficiency and management of laboratory supplies. Examples include:

- Solomon Islands: operates a nationally managed supply chain system, where PHC facilities funnel their supply orders to the national level through their provinces.
- Philippines: local governments submit their usage data to the national level, which is used to plan centralized procurement each year.

Supply chain management challenges that exacerbate delays and contribute to inconsistent availability of essential laboratory materials were reported by six countries:

- Prolonged stock-outs: shortages of equipment spare parts, reagents and kits, and other consumables can last more than 6 months.
- Short-expiry reagents: sometimes countries receive delivery of reagents with limited shelf life which leads to waste and inefficiencies.
- Service disruption: lack of supplies forces facilities to turn away patients or discard collected samples. A frontline health worker reported that due to a lack of reagents and consumables, *“…patients are sometimes turned away or collected patient samples are discarded”.*
- Use of suboptimal diagnostics: the supply disruptions may also lead to the use of expired and compromised reagents or less sensitive diagnostic methods. One frontline level respondent reported the interchangeable use of the more sensitive GeneXpert and the less sensitive sputum smear microscopy for TB diagnosis despite the latter being suboptimal.
- Geographic and environmental barriers: countries which consist of complicated archipelagos (e.g., Philippines, Solomon Islands), face additional logistical challenges. Moving supplies from centralized stores to remote PHC facilities is hindered by flooded roads, rough seas or unpredictable weather conditions.

5. Sample referral and transportation systems

The functionality and effectiveness of sample transportation systems differ across countries in the region.

- Malaysia and China: reported effective well-functioning sample transport systems
- Other countries: reported that they are working towards improving their systems. They have national guidelines on sample handling, packaging, and transportation.

However, seven countries reported sample transport and referral challenges that include:

- Funding dependency: most sample transportation activities are funded through vertical disease programs. The Global Fund supports sample transportation for disease programs like HIV, TB and malaria, including developing sample collection and referral protocols and sample transportation from PHC centres to district and provincial hospital laboratories.
- Infrastructure gaps: rural areas and other hard-to-reach areas have limited transport options, staff shortages, and a lack of infrastructure making sample transportation challenges.
- Use of non-ideal transport methods: it was reported that non-ideal transport systems that are not customized for sample transport like tuk-tuks and motorbikes are routinely used. Without appropriate sample transport packaging this can potentially compromise the quality of samples.

6. Laboratory information systems and data management

Some countries have made strides in implementing laboratory information systems (LIS) and data management:

- China: reported extensive digital connectivity across the country, facilitating result reporting. One national-level health officer stated, “*In China, everything is quick, with electronic results within 1 day. There is also further reduction of delays through centralisation*”.
- Cambodia: uses the CAMLIS system, which is supported by WHO and the MOH.
- Malaysia: reported that work towards digitalization of the health services to improve the data collection and sharing is ongoing with pilot studies on a web-based system for information sharing between facilities currently running in some localities.

Despite some of these advancements, all countries reported challenges with LIS and data management:

- High costs of technology: the expense associated with introducing and using new technologies put them out of reach for many countries.
- Limited information technology infrastructure: most countries have limited internet connectivity and inadequate information technology equipment and skills. Hence, LIS is not fully implemented across the laboratory network.
- Fragmented data management systems: different disease programs often have their own reporting systems, developed independently or over time. This creates fragmentation and makes it difficult for the systems to communicate or share data efficiently. One NGO expert described the country’s laboratory data management systems as “*different reporting systems for diseases programs that appear disparate, unharmonized, and not interoperable. Limited connectivity and inadequate IT equipment and skills prevent full utilization of health information systems*.”
- Reliance on manual paper-based systems: some countries use paper-based systems, which are prone to error and inefficiencies, while others use both a paper-based system and a parallel electronic system, creating a complex and labour-intensive duplicate system that confuses staff. Data in paper-based system are not easily accessible or are lost before reaching clinicians or patients. One country reported that *“If a patient visits a PHC centre and can’t get tests they want and are referred to the* *district, there is no way for PHC centre to get access to the same information at the district hospital”.*
- Use of unsecure communication channels: in the absence of robust LIS, social media communication channels (WhatsApp, Telegram, Messenger, etc.) are sometimes used informally to share patient results between PHC and higher-level laboratories. One frontline health worker shared that, “*We have a WhatsApp group where the sputum results for TB are sent so clinicians can assess them without having to travel to the laboratory.”*

### Limited community and individual engagement

1. Affordability of services

All countries reported some challenges related to the affordability of laboratory testing and the cost burden on patients:

- Unfunded services: it was reported that patients face expensive out-of-pocket costs for services not funded by their governments, health insurance or vertical programs.
- High indirect costs: when services are not available locally, patients also face additional costs such as transportation, overnight accommodation, and loss of income away from work due to long distances travelled to access referral health services. In one country, it was reported that patients travel long distances to district hospitals to access HIV and hepatitis testing because they are not available at the PHC level.
- Expensive private sector services: disruptions in public testing services also force patients to pay high costs to access services from the private sector where they are charged upfront due to delayed reimbursements. One provincial-level expert noted, *“Reimbursement can take a year after the services, which doesn’t work if a private facility depends on the income to run the facilities and offer services”*.

2. Accessibility of services

Seven countries reported challenges related to the accessibility of laboratory services, particularly in remote and underserved communities:

- Geographic isolation: some remote communities cannot easily access the nearest PHC facility due to distances or location of the community (mountains, forests, islands, rivers, etc.).
- Cultural and religious constraints: in some regions women will not seek healthcare or decline to receive treatment from male health workers for religious or cultural reasons. However, there are more male than female specialist health workers, confounding the situation.
- Unfavourable facility operating hours: conflict can occur between operating hours and other business hours so patients cannot visit laboratories because they are at work. There are also long waiting times at primary health centres facilities due to staffing challenges. In addition, most rural and remote health facilities are manned by a single staff member and usually closes every time the staff member is on leave or unwell.
- Limited testing services in remote areas: some remote PHC facilities offer a limited range of tests but are also prone to interruption of services due to supply chain, staffing or equipment issues. These discourage patients from seeking laboratory services. One frontline health worker remarked, *“Patients in remote areas are usually low socio-economic class. Services in these areas are limited and usually not free, so people may have to travel to find either adequate or free/cheaper services. However, the cost of travel can be an issue”*. Another frontline worker commented, *“If a mother has suspected syphilis, and blood is taken and referred for testing, it is unlikely that the patient would return for the test results due to the distance, or economic reasons”*.
- Lack of health education: lack of health information and education often results in patients waiting until they are extremely sick before seeking medical attention. At this stage, they may require specialist services since the PHC level may be unable to test and treat them. Some people may be unaware of the availability of free services, further delaying care.

3. Stigma and discrimination

Some countries are putting measures in place to address stigma and discrimination and improve access for marginalized populations:

- Mongolia: NGOs collaborate with the MOH to conduct training and focus group meetings with healthcare workers, students, legal organizations, law enforcement personnel, and communities to dispel myths and stereotypes to reduce stigma about key populations. They bring sexual health testing services closer to groups that will otherwise not seek services in an inclusive environment by conducting outreaches in hot spots (e.g., nightclubs).
- Lao PDR: mobile HIV testing for female sex workers, health screening for isolated communities and health education by nurses increase access to health and laboratory services for the hard-to-reach communities.
- Cambodia: employs local staff who speak minority languages to ensure that remote and isolated communities receive laboratory testing and other health services in their language, which encourages community ownership of services.

Despite these efforts, challenges still exist and were reported by four countries:

- Stigma and confidentiality breaches: the stigma associated with infectious diseases and breach of confidentiality causes patients to be shunned by their communities or lose jobs. Fear of incarceration results in reduced uptake of health services and diagnostic testing among key populations.
- Uneven legal protections: some participants noted that although HIV patients are often legally protected against stigma and discrimination, other communicable disease patients still face discrimination (e.g., from employers).
- Clinical guidelines are not inclusive: the absence of clinical guidelines and laboratory reference ranges for transgender persons reduces the quality of health and wellness services received.
- Self-stigma and delayed care: it was reported that men are sometimes embarrassed to access clinics or laboratories for STI services until they are visibly sick, and sometimes they go directly to the pharmacy for antibiotics.
- Limited awareness of support services: lack of awareness about services available, (e.g., legal and counselling) hampers health-seeking behaviour of communities.
- Community engagement

4. Community engagement

Five countries reported meaningful community involvement in healthcare decision-making and service delivery at the PHC level:

- Cambodia: uses participatory health governance models (e.g., the Health Centre Management Committee, the Village Health Support Group, and the Village Health Volunteers) that further partnerships between communities and government and strengthen the linkage between the community and the health centres. Local community representatives and providers jointly govern health facilities through committees.
- Malaysia: leverages its strong community-based maternal and child health services to address communicable diseases and emerging NCD public health challenges at the PHC level.
- PNG: the MOH enters into agreements with FBOs in the country to support service delivery in rural and remote areas.
- Lao PDR and PNG: community engagement and outreach, education and support activities by community health workers (CHW) and volunteers were instrumental in controlling malaria.

However, six countries reported barriers to effective community engagement:

- Limited community-led initiatives: a lack of or minimal existence of established community-led initiatives, and monitoring and support activities to optimize the participation of patient groups and patient support groups.
- Health education gaps: poor awareness of available free services at PHC sometimes leads to patients rushing to expensive private sector where the services are usually readily available.
- Health workers knowledge gaps on emerging diseases: a lack of knowledge about less common or new and emerging infectious diseases (e.g., Mpox) hampers the quality of service provided at the PHC level.
- Fragmented CHW efforts: it was also noted that CHW and volunteers usually focus only on funded programs and hence deliver parallel activities to PHC services, leaving other PHC activities and disease areas under-resourced.
- Licensure confusion: licensure requirements for NGOs, CBOs and FBOs are usually fragmented, making the status of NGOs, CBOs, and FBOs that conduct screening tests and offer other PHC services unclear, creating confusion among the patient groups and communities they were set up to reach and serve.

### Persistent socio-economic barriers

1. Funding health services

Most countries reported that they offer free (or nominal fee) healthcare and laboratory testing services through national health insurance or other national funding systems. Private health providers also complement the public services, and patients pay out-of-pocket or through insurance.

However, health services funding challenges were also noted by five countries:

- Heavy donor dependency: several disease programs (e.g., TB, HIV, and malaria) in most countries rely heavily on donor funding (e.g., The Global Fund) for the supply of equipment, reagents, and consumables.
- Sustainability concerns: program budget constraints and lack of sustainability beyond the funding cycle causes many patients to incur out-of-pocket costs. In one country the funds committed towards TB testing and treatment as part of the Global Fund contributory agreements were exhausted during the COVID pandemic causing delays in procurement of drugs, testing reagents, and consumables. The government eventually turned to the World Bank, another international funding partner to bridge the funding gap.

2. Multisectoral and cross-cutting issues

Multisectoral and cross-cutting challenges were reported by three countries:

- Fragmentation of vertical disease programs: most vertical disease programs in the region are usually fragmented, without centralized government support. This leads to duplication of efforts, gaps in service delivery, and poor integration with broader health systems.
- Regulatory and licensure restrictions: it was noted that regulation and licensure restrictions also hamper the expansion of services offered at the PHC level. In one country, NGO-run clinics keen to expand services face regulatory challenges: *“There was a CBO laboratory that stopped operations because their technologist resigned. A licensed Medical Laboratory Technologist is part of licencing requirements, and without a technologist, the laboratory could not continue operations.”*
- Limited private sector engagement: program funding does not support private sector participation in health programs. For example, training costs for private healthcare providers are not covered, failing to incentivize private sector participation, thereby limiting patient access to services. This also results in poor treatment outcomes and a lack of reporting for mandatory case notification, weakening disease surveillance and response systems.

3. Potential outbreak response

During the evaluation, three countries reported challenges related to their readiness for potential outbreaks:

- Lack of emergency planning: it was reported that there was often a lack of or fragmented contingency plans for emergencies, with responses to emergencies being planned only when encountered.
- COVID-19 pandemic-induced challenges: countries noted that the COVID-19 pandemic resulted in service reallocation and reduced access to routine services. Population coverage of communicable disease services was impacted, resulting in a scaling back of services, e.g., TB services. Staff were reallocated to COVID-19 preparedness, screening, diagnosis, and treatment services. Communicable disease clinics and hospitalization services, (e.g., TB clinics and TB isolation wards) were repurposed to house suspected and confirmed COVID-19 cases. In most countries, patients could not visit health centres for routine PHC and preventive medicine services during the pandemic-instituted mandates and restrictions.

## Discussion

### Weaknesses in PHC systems

Several persistent challenges that contribute to weaknesses in the PHC systems were noted. Key issues included limited access to quality laboratory services, fragmented vertical programs, workforce shortages, supply chain problems, inadequate sample transport and referral systems, and the absence of integrated LIS and data systems.

In many countries, several vertical disease programs, focused on specific diseases or conditions, are being implemented through development partnerships. These programs are often favoured by donors due to their clearly defined objectives, targeted impact, and ability to bridge healthcare funding gaps in the short to medium term [4, 7]. They often come with dedicated staff, specialized training, and essential resources. Some platforms funded through vertical programs (such as GeneXpert analyzers procured via Global Fund initiatives for TB), also have diagnostic potential for other diseases such as STI and oncology.

Despite their benefits, vertical programs present several challenges [4, 7, 8]. They can duplicate activities across different health initiatives, creating inefficiencies and increasing costs. Without adequate coordination, programs may target the same populations separately, undermining the delivery of comprehensive, people-centred healthcare. Furthermore, many of these programs focus narrowly on disease-specific outcomes rather than supporting broader health system strengthening. As a result, their sustainability beyond donor funding cycles is uncertain. Most sample referral systems, for example, are financed by these programs and risk collapse once external funding ends.

There is often untapped potential in optimizing the use of laboratory equipment already available through vertical programs. Expanding the test menu of devices such as GeneXpert analyzers to include additional conditions like syphilis or HIV could increase access to diagnostics. However, high costs for reagents, lack of flexible funding, and donor-imposed restrictions limit broader utilization. On the other hand, WHO and partners (e.g., USAID) are helping to address some of the challenges like underfunding of QA activities by providing technical support to strengthen national QA systems. A notable example is the WHO-supported malaria microscopy QA program, which improved microscopist competency using slide banks and external assessments, demonstrating effectiveness at a relatively low and sustainable cost [16].

Workforce shortages at PHC level significantly hinder the quality and availability of laboratory services. Staff are often unavailable to collect, package, and transport specimens to referral laboratories. The shortage of biomedical engineering technicians also impacts timely equipment maintenance and repair, leading to extended downtimes. In many facilities, the absence of trained laboratory personnel forces testing to be done by unqualified clinical staff or CHW, compromising quality. Task shifting, if supported by adequate training, mentoring, and supervision, can serve as a temporary solution. However, working conditions in rural and remote PHC facilities remain poor, deterring qualified professionals from accepting or remaining in these posts [17]. In some cases, staff who were reassigned as part of the COVID-19 pandemic response have not returned to their original roles, further contributing to ongoing personnel shortages [17, 18].

Supply chain disruptions, aggravated by the COVID-19 pandemic, continue to affect PHC operations. Supplies depleted during the pandemic have not been replenished due to global shortages, rising costs, procurement delays, and local supplier failures. Weak procurement and supply management systems further limit the consistent availability of diagnostic materials. Although some countries have reviewed their national testing algorithms to cope with supply issues, substituting highly sensitive diagnostics with less sensitive alternatives compromises diagnostic accuracy and patient safety. Such practices are not sustainable and undermine public trust in the health system.

Combined, workforce shortages, supply issues, and inefficient sample referral systems contribute to delayed test results. These delays discourage clinicians from requesting laboratory tests, lead to loss of patient follow-up, and result in reliance on empirical or syndromic treatment. This, in turn, raises the risk of poor clinical outcomes and contributes to public health threats such as antimicrobial resistance. Implementing integrated laboratory information systems (LIS) that serve all disease programs can improve result management, turnaround time, and data use. Unfortunately, many countries face technical and financial barriers to deploying such systems. In their absence, some facilities rely on insecure social communication channels to transmit test results. While this improves communication speed, it raises serious concerns around patient privacy and data confidentiality.

### Limited community and individual engagement

Communities served by PHC facilities, especially rural households and populations experiencing poverty, are often vulnerable to financial hardship and catastrophic health expenditures. This study confirmed that even individuals entitled to free care through social protection programs may hesitate to access services due to lack of information, transportation costs, or potential loss of income from missing work.

Lack of health education and promotion contributes significantly to poor health-seeking behaviour. Without knowledge of available preventive and curative services, communities may delay or forgo seeking care altogether. Improving health education and promotion at the community level can help address these barriers. Effective strategies include the use of visual aids and technology such as text messaging and social media to strengthen community engagement and communication systems.

Stigma, defined as stereotyping and marginalizing individuals or groups based on perceived characteristics, can further deter people from seeking care [19]. It can negatively impact both service uptake and health outcomes for stigmatized and hard-to-reach groups. For example, in this study it was noted that individuals with infectious diseases may delay visiting health facilities or undergoing laboratory testing due to fear of being labelled or excluded, potentially leading to wider community transmission and spread of infectious diseases.

Community engagement is critical in overcoming most of these challenges. It involves consulting and collaborating with communities on problems and challenges, decisions, and solutions that affect them, allowing them to actively contribute to health system strengthening and service design [20, 21]. CHW who are trained in the basics of preventive care, POC testing, and result interpretation are central to the success of PHC programs. Malaria control initiatives, for example, have shown improved case management and stronger community participation through advocacy, awareness, and prevention activities led by CHW [22].

### Persistent socio-economic barriers

Adequate funding for health services, particularly PHC services, is essential for improving equity and ensuring access for all population groups. It plays a critical role in helping countries achieve UHC. In contrast, underfunded health systems leave vulnerable and high-risk groups exposed to catastrophic out-of-pocket expenditures, deepening poverty and health inequities [23]. However, in many countries, national health budgets are distributed unevenly across different levels of the health system. This often leaves poorer provinces and local governments unable to provide essential services due to budget constraints, further exacerbating disparities in health outcomes [24].

Addressing health inequities and strengthening PHC requires a multisectoral approach. Many structural and social determinants of health and the effectiveness of diagnostic services lie beyond the control of the health sector alone [7]. Responsibility must be shared with other government agencies, development partners, communities, and relevant stakeholders. Collaborative approaches that bring together various stakeholders can support the integration of vertical disease programs into existing PHC activities and improve laboratory testing services [16].

The COVID-19 pandemic underscored the importance of such multisectoral collaboration. It revealed critical gaps in surveillance, monitoring, and early response systems needed to detect and respond to disease outbreaks [17, 25]. Global agencies such as WHO and the One Health Quadripartite have since advocated for stronger coordination across sectors, including animal health, environmental services and the private sector to prevent and control zoonotic and neglected tropical diseases [23].

The likelihood of future global pandemics remains high due to several factors [19]. Increased global connectivity allows diseases to spread rapidly across borders, while climate change is accelerating the emergence of new disease threats, particularly zoonoses. Countries must act on the lessons learned from COVID-19 by establishing robust pandemic preparedness plans. These should include the ability to quickly mobilize trained epidemiologists and disease control specialists to lead coordinated outbreak responses [25].

## Limitations and strengths of the study

There were three potential limitations of this evaluation. Selection bias might have occurred since the subject matter experts were drawn from a non-random sample selected by each country’s MOH in collaboration with the WHOCO. The participants might have given responses and perspectives perceived to be favourable to their superiors and may not represent those of other key personnel or organizations that were not selected. However, MOH and WHOCO thoroughly understand the PHC and key opinion leaders in their countries. Hence, we believe they selected the most appropriate experts. These participants included experts at all health system levels, community representatives, patients, and representatives of NGOs, CBOs, and FBOs. Second, due to the limited time, private sector participants were not included, and their views and experiences were not captured. The private health sector has grown rapidly in recent years. However, much of the population in the region accesses PHC services from the public system, and/or development partner-funded organizations and their views and perspectives informed the findings of this evaluation. Finally, the multi-cultural nature and language diversity of the region required the utilization of translators in some countries, potentially introducing barriers to interpreting and conveying participant experiences fully and accurately. To mitigate this, bilingual WHO Country Office officials and/or professional translators provided the translation services where required.

Despite these limitations, this study provides timely insights and perspectives from experts across multiple levels of the health system on the status, capacity, and infrastructure of laboratory services at the PHC level in eight middle-income countries within the Western Pacific Region. It underscores systemic challenges that hinder access to essential laboratory services, which may also be relevant to other middle-income countries with comparable demographic and health system characteristics. These findings can support policymakers in developing targeted recommendations and solutions to reduce disparities in access and support progress toward UHC.

## Conclusion

This evaluation assessed the status, capacity, and infrastructure of the laboratory systems and identified systemic challenges affecting access to essential laboratory services at the PHC level in eight middle-income countries within the WHO Western Pacific Region. Use of POC testing and sample transport system for referral of samples to higher tier testing centres (funded mainly through vertical disease programs) enhances access and availability of laboratory services at the PHC level. Community engagement strengthens linkages between communities and health centres and enhances activities to combat stigma and discrimination. Availability of national health insurance programs enables free laboratory testing for most of the population.

The main challenges were categorized into three thematic areas: (a) weaknesses in PHC systems; (b) limited community and individual engagement; and (c) persistent socio-economic barriers. The list of challenges is not exhaustive but a snapshot of the barriers hindering access to laboratory services at the PHC level in the region. Any potential solutions and recommendations suggested should be customized for each country’s context in collaboration with the MOH and PHC experts before implementation using a stepwise approach.

## Supplementary Information

Additional file1. Table: Interview guides and corresponding participants groups. Interview guide 1: National level Interview guide. Interview guide 2: Provincial level Interview guide. Interview guide 3: Frontline Health worker Interview guide. Interview guide 4: Patients and community leaders Interview guide

## Data Availability

See supplementary information for questionnaires used in data collection.

## Abbreviations

CBO: Community-based organizations
CHW: Community health workers
FBO: Faith-based organizations
HIV: Human immunodeficiency virus
Lao PDR: Lao People’s Democratic Republic
LIS: Laboratory information systems
LMIC: Low- and middle-income countries
MOH: Ministry of Health
NCD: Noncommunicable diseases
NGO: Non-governmental organizations
PCR: Polymerase chain reaction
PNG: Papua New Guinea
PHC: Primary healthcare
POC: Point of care
QA: Quality assurance
TB: Tuberculosis
UHC: Universal health coverage
WHO: World Health Organization
WHOCO: WHO Country offices
