# Supplementary material for: A qualitative evaluation of access to essential laboratory services for communicable diseases at the primary health care level in the Western Pacific Region
Source: Trop Med Health. 2025 Nov 7;53:156. doi: 10.1186/s41182-025-00797-3 (PMC12595625; doi:10.1186/s41182-025-00797-3)
Supplement: Supplementary file 1 — Additional file1. Table: Interview guides and corresponding participants groups. Interview guide 1: National level Interview guide. Interview guide 2: Provincial level Interview guide. Interview guide 3: Frontline Health worker Interview guide. Interview guide 4: Patients and community leaders Interview guide [file 41182_2025_797_MOESM1_ESM.docx]

# **Additional Information**

## **Additional information file 1: Table: Interview guides and corresponding participants groups**

| Interview guide | Participants |
| --- | --- |
| National level interview guide | - WHO technical representatives - National level health officers - National level representatives of CBOs, NGOs and FBOs |
| Provincial level interview guide | - Provincial level health officers - Provincial level representatives of CBOs, NGOs and FBOs |
| Frontline health workers interview guide | - District level health workers - Frontline health workers - Frontline level representatives of CBOs, NGOs and FBOs |
| Patients or community leaders interview guide | - Patients - Patient representatives - Community leaders |

Legend:

- WHO: World Health Organization
- CBOs: Community-based organisations
- NGOs: Non-governmental organisations
- FBOs: Faith-based organisations

## **Additional information file 2: Interview guide 1: National level Interview guide**

**Name of the interviewer ___________________________________________________________**

**Name, age, sex, and occupation of the interviewee ______________________________________**

**Place and date of interview _________________________________________________________**

Introduction

Dear Interviewee, this interview is being conducted to understand better access to primary health care focusing on laboratory services in the country. Your insights and feedback will contribute to our understanding and help shape the strategies for improving health outcomes for all, particularly the unreached populations.

Interview Structure

This semi-structured interview comprises 10 key questions, with a few probing questions, which we will use only if required. The questions are open-ended to encourage you to share your thoughts in-depth. The interview should take approximately 1 hour.

| **Key Questions** | **Probing Questions** |
| --- | --- |
| 1. How is access to primary health care, specifically lab services, currently structured at the national level? | - Can you describe the existing infrastructure?  - What policies guide the provision of lab services?  - How is the system financed? |
| 2. What significant challenges are faced in providing laboratory services at the primary care level? | - Are there any specific barriers related to equipment or supply chains?  - How is the workforce for these services recruited and trained? How is testing competency determined? Continuous education programs?  - How are services monitored and evaluated? |
| 3. What are the socioeconomic barriers to access to laboratory services currently, and how are they being addressed? | - What initiatives have been implemented to improve affordability?  - How are geographic barriers handled?  - Are there strategies in place to address cultural, social or linguistic challenges? |
| 4. How does the national health system ensure the quality of lab services? | - Are there quality assurance protocols in place?  - How is technology leveraged to improve the quality of services? Is there an integrated LIS?  - How is the quality of services monitored and improved?  -How are test kits chosen and purchased? Is there test kit ‘stock outs’?  How are testing strategies and testing algorithms selected?  -Are primary healthcare testing sites supervised by higher tier laboratories?  -Do PHC’s participate in EQAS? Is it national or international? |
| 5. How are laboratory services integrated into broader health services? | - How are lab results used to inform treatment plans?  - What role do lab services play in preventive health care?  - How is patient information shared across services? |
| 6. How do lab services cater to different population groups, especially unreached groups? | - How is equity in access ensured?  - Are there special programs or strategies for specific groups?  - How are challenges related to reaching unreached groups handled? |
| 7. How is the community involved in the decision-making and implementation of laboratory services? | - What avenues for community input exist?  - How is community feedback incorporated?  - What roles do community health workers play? |
| 8. How are potential public health emergencies planned for lab services? | - Are there contingency plans in place?  - How is the lab capacity scaled up during emergencies?  - How does the system adapt to changing needs?  -Describe the outbreak reporting system?  Are there any systems to support disease surveillance? |
| 9. What are the current strategies to foster multisectoral action to overcome barriers to primary care, mainly lab services? | - How are partnerships with other sectors built and maintained?  - What role do non-health sectors play?  - Can you share an example of a successful multisectoral initiative? |
| 10. What is your vision for the future of primary health care, mainly laboratory services, in the country? | - How would you like to see lab services evolve?  - What changes would you like to see in policy and practice?  - What actions are necessary to achieve this vision? |

**Disclaimer**: This interview is confidential, and your information will be anonymised. Please feel free to ask any questions or seek clarification on anything unclear.

**Note**: As is the rule with guides, skipping some questions and inventing new ones based on the need of the day is not just allowable but encouraged if most (if not all) of the objectives are met. This is not a survey form or a checklist but rather a guide. Also, generating new questions and dropping some may depend on ‘data’ saturation.

## **Additional information file 3: Interview guide 2: Provincial level Interview guide**

**Name of the interviewer ___________________________________________________________**

**Name, age, sex, and occupation of the interviewee ______________________________________**

**Place and date of interview _________________________________________________________**

Introduction

Dear Interviewee, we are conducting this interview to better comprehend the accessibility, quality, and challenges of primary health care, specifically laboratory services, at the provincial level. Your insights will be instrumental in our efforts to improve health outcomes, particularly for unreached populations.

Interview Structure

This semi-structured interview comprises 10 key questions, with a few probing questions, which we will use only if required. The questions are open-ended to encourage you to share your thoughts in-depth. The interview should take approximately 1 hour.

| **Key Questions** | **Probing Questions** |
| --- | --- |
| 1. How is the primary healthcare system, mainly laboratory services, structured at the provincial level? | - Could you describe the current infrastructure?  - What are the critical local health policies that guide lab services?  - How is the system financed? |
| 2. What are the specific challenges in providing laboratory services at the primary care level in your province? | - Are there any specific barriers related to logistics or supply chains?  - How is the recruitment and training of lab staff handled? How is testing capacity assessed? And continuous professional education?  - Are there issues with staff retention? |
| 3. In your opinion, what are the main socioeconomic barriers that affect access to laboratory services? | - Are services affordable for all residents?  - Are geographical barriers significant in your province?  - Do linguistic or cultural issues affect access?  -Are there situations where patients do not find the testing process acceptable to their needs or preferences? |
| 4. How does the provincial health system ensure quality lab services are delivered? | - Are there standard protocols or quality control measures in place?  - How is technology leveraged to improve service quality?  - Is there a system to handle complaints or feedback?  -Do you participate in EQAS? For which tests? |
| 5. How are laboratory services integrated into broader health services at the provincial level? | - How are lab results utilised in clinical decision-making?  - How do lab services contribute to preventive health care in your province?  - How is patient information coordinated across different services?  -How are PHC testing sites supervised? |
| 6. How are unreached or vulnerable populations catered to when providing lab services? | - How is equity in access to services ensured in your province?  - Are there special initiatives or strategies for specific population groups?  - How are challenges related to reaching these groups overcome?  - How are the testing needs and preferences of unreached or vulnerable populations incorporated into the testing process? |
| 7. What role does the community play in the decision-making and implementation of laboratory services? | - Are there mechanisms for gathering and integrating community feedback?  - How are community health workers utilised?  - Are there partnerships with community-based organisations? |
| 8. How are potential public health emergencies managed with laboratory services? | - What contingency plans are in place?  - How is lab capacity expanded during emergencies? - How does your province handle a surge in demand for lab services?  -Describe the outbreak reporting system |
| 9. How are multisectoral strategies used to overcome barriers to primary care, including laboratory services? | - How does your province partner with non-health sectors?  - Can you share examples of successful multisectoral initiatives?  - What role do local governments play in these initiatives? |
| 10. How do you envision the future of primary healthcare, mainly laboratory services, in your province? | - What improvements would you like to see?  - What changes are needed in policy or infrastructure?  - What actions are necessary to realize this vision? |

**Disclaimer**: This interview is confidential, and your information will be anonymised. Please feel free to ask any questions or seek clarification on anything unclear.

**Note**: As is the rule with guides, skipping some questions and inventing new ones based on the need of the day is not just allowable but encouraged if most (if not all) of the objectives are met. This is not a survey form or a checklist but rather a guide. Also, generating new questions and dropping some may depend on ‘data’ saturation.

## **Additional information file 4: Interview guide 3: Frontline Health worker Interview guide**

*Frontline Health workers could be doctors, nurses, laboratory technicians and others.*

**Name of the interviewer ___________________________________________________________**

**Name, age, sex, and occupation of the interviewee ______________________________________**

**Place and date of interview _________________________________________________________**

Introduction

Dear Interviewee, we want to understand your experiences and perspectives as a frontline worker interacting directly with patients. Your insights into primary health care's provision, challenges, and opportunities, especially laboratory services, are crucial.

Interview Structure

This interview comprises 10 key questions, with a few probing questions, which we will use only if required. We encourage detailed responses to provide deep insights. The interview should take around an hour.

| **Key Questions** | **Probing Questions** |
| --- | --- |
| 1. Can you describe your role in providing primary healthcare services, mainly laboratory services? | - What are your key responsibilities?  - What is the workflow of providing lab services?  - How do you interact with patients and other health professionals? |
| 1. What challenges do you face in providing effective laboratory services to patients? | - Do you experience equipment or supply shortages?  - Are there difficulties in conducting specific tests?  - Do you encounter challenges with time management?  -Did you receive adequate training? Was your testing competency assessed before you began testing patients?  - Are you receiving any continuous professional development or training? |
| 1. What are the significant barriers patients face in accessing laboratory services? | - Are certain patient groups more affected than others?  - How does affordability affect access to services?  - Are there issues with geographic accessibility or clinic schedules? |
| 1. How is the quality of laboratory services ensured in your role? | - How do you maintain the reliability of test results?  - What procedures are in place for quality control?  - How do you manage equipment maintenance? |
| 1. How are laboratory services integrated into the overall patient care pathway? | - How are lab results communicated to patients?  -How long does it take for test results to be reported to the patient?  - How are lab services coordinated with other healthcare services?  - How do you ensure a seamless transition for the patient through various services?  - For tests not offered by facility do you refer patients or samples? |
| 1. How does your work help reach unreached populations, especially regarding laboratory services? | - What specific strategies or initiatives are you part of?  - How are services adapted for different population groups?  - How is continuity of care ensured for these groups?  - How are the needs and preferences of unreached populations incorporated into the testing process? |
| 1. Can you describe any community involvement in the decision-making or implementation of laboratory services? | - How does the community provide feedback or input?  - How are community health workers involved?  - Do you have examples of successful community partnerships? |
| 1. What measures are in place to handle the increased demand for lab services during public health emergencies? | - How do you manage a sudden increase in patient numbers?  - How are supply chains maintained during emergencies?  - Are there specific emergency protocols to follow?  -If you suspect an outbreak where do you report to? |
| 1. Can you share examples of multisectoral initiatives that have improved access to lab services? | - How do non-health sectors contribute to these initiatives?  - How do such initiatives impact your work?  - What has been the response from patients? |
| 1. What improvements would you like to see in providing primary healthcare and laboratory services? | - What changes would be beneficial to your work?  - What could improve patient outcomes?  - How do you envision the future of lab services in primary care? |

**Disclaimer**: This interview is confidential, and your information will be anonymised. Please feel free to ask any questions or seek clarification on anything unclear.

**Note**: As is the rule with guides, skipping some questions and inventing new ones based on the need of the day is not just allowable but encouraged if most (if not all) of the objectives are met. This is not a survey form or a checklist but rather a guide. Also, generating new questions and dropping some may depend on ‘data’ saturation.

## **Additional information file 5: Interview guide 4: Patients and community leaders Interview guide**

*These subject matter experts could be from among the rural poor and urban poor areas, identified after interviewing the provincial health officers and frontline health workers using snowballing. If the recommended person cannot understand or speak in English, the person who recommends may help with the translation, or a local officer in WHO Country Office who knows their language may need to translate.*

**Name of the interviewer ___________________________________________________________**

**Name, age, sex, and occupation of the interviewee ______________________________________**

**Place and date of interview _________________________________________________________**

Introduction

Dear Interviewee, we are interested in hearing about your experiences and perspectives regarding primary health care, mainly laboratory services, in your community. Your insights are invaluable in improving access to and quality of these services.

Interview Structure

This interview comprises 10 key questions, with a few probing questions, which we will use only if required. We encourage detailed responses to provide deep insights. The interview should take around an hour.

| **Key Questions** | **Probing Questions** |
| --- | --- |
| 1. Can you describe your (or your communities’) experiences accessing primary healthcare services, specifically laboratory ones? | - How easy or difficult is it to access these services?  - What types of lab services do you usually require?  - How satisfied are you with the current services?  - Do you find the testing process meets your needs and preferences? |
| 2. What challenges do you face in accessing effective laboratory services? | - Do you experience issues with affordability or transportation?  - Are there challenges with clinic schedules or waiting times?  - Do you face any cultural, language or communication barriers? |
| 3. How do you perceive the quality of laboratory services available? | - How reliable do you think the test results are?  - Do you feel comfortable during the process?  - How is the follow-up care after tests are done? |
| 4. How well are laboratory services integrated with other healthcare services you receive? | - How are lab results communicated to you?  How long does it take to receive your results?  - Is there coordination between lab services and other healthcare services?  - How seamless is your transition through various services? |
| 5. What measures are taken by the healthcare system to reach the unreached population in your community, particularly regarding laboratory services? | - Are you aware of any specific strategies or initiatives (e.g., specimen transport services, outreach services)?  - How have these strategies affected you or your community?  - How is continuity of care ensured for these groups? |
| 6. Can you describe your community's involvement in the decision-making or implementation of laboratory services? | - How does the community provide feedback or input?  - Are community health workers involved?  - Can you share examples of successful community partnerships?  - Have you been tested in the community, I.e., outside of a laboratory? |
| 7. How are laboratory services managed during public health emergencies (e.g., during the COVID-19 pandemic)? | - How is the increased demand for services handled?  - Are there changes in the availability of lab services?  - How is the community informed about these changes? |
| 8. Are there any multisectoral initiatives that have improved access to lab services in your community? | - What non-health sectors are involved in these initiatives (e.g., community leaders who are active in other sectors)?  - How have these initiatives impacted you or your community?  - How would you evaluate the success of these initiatives? |
| 9. Are you aware of digital technologies in your healthcare system, mainly laboratory services? | - How does this affect your access to services?  - What is your experience using these technologies?  - Do you face any barriers to using digital technologies? |
| 10. What improvements would you like to see in providing primary healthcare and laboratory services in your community? | - What changes would benefit you or your community?  - What could improve your satisfaction with the services?  - How do you envision the future of lab services in primary care? |

**Disclaimer**: This interview is confidential, and your information will be anonymised. Please feel free to ask any questions or seek clarification on anything unclear.

**Note**: As is the rule with guides, skipping some questions and inventing new ones based on the need of the day is not just allowable but encouraged if most (if not all) of the objectives are met. This is not a survey form or a checklist but rather a guide. Also, generating new questions and dropping some may depend on ‘data’ saturation.
